# The Differences in the Safety and Tolerability of Immune Checkpoint Inhibitors as Treatment for Non–Small Cell Lung Cancer and Melanoma: Network Meta-Analysis and Systematic Review

**DOI:** 10.3389/fphar.2019.01260

**Published:** 2019-10-24

**Authors:** Qing-Qing Chai, Jiang-Yang Du, Jun Zhu, Bin Wu

**Affiliations:** ^1^Department of Pharmacy, Shanghai Chest Hospital, Shanghai Jiao Tong University, Shanghai, China; ^2^Medical Decision and Economic Group, Department of Pharmacy, Ren Ji Hospital, School of Medicine, Shanghai Jiao Tong University, Shanghai, China

**Keywords:** immune checkpoint inhibitors, non–small cell lung cancer, melanoma, network meta-analysis, treatment-related adverse events

## Abstract

**Background:** Immune checkpoint inhibitors (ICIs) have evolved for the treatment of solid tumors. In addition to the efficacy of ICIs for cancer, the adverse events (AEs) of ICIs are also noteworthy for gradually more extensive clinical use.

**Objective:** To conduct a systematic review and network meta-analysis to evaluate the treatment-related AEs that occurred in clinical trials using different kinds of ICIs, to explore the differences in AEs among ICIs for treating non–small cell lung cancer (NSCLC) and melanoma, and to compare select immune-related AEs.

**Methods:** PubMed, EMBASE, Cochrane Library, ClinicalTrials.gov, and other available sources were systematically searched for published reports up to January 1, 2019. Two reviewers independently selected reports about phase II/III randomized controlled trials to compare among ICIs and between ICIs and chemotherapy. After the bias assessment of all included trials, a Bayesian network meta-analysis was performed. The primary outcomes were any-grade and high-grade treatment-related AEs from all ICIs. The secondary outcomes were AEs in patients with NSCLC and melanoma and the presence of the select AEs pneumonitis/pneumonia and colitis.

**Results:** Eighteen randomized controlled trials containing 11,223 patients with NSCLC or melanoma were included. A total network meta-analysis was conducted. The meta-analysis showed that atezolizumab 1,200 mg and pembrolizumab 2 mg/kg every 3 weeks were generally more tolerable than other ICIs. ICI combined with chemotherapy might suggest a higher risk of treatment-related AEs than monotherapy with a single ICI, except durvalumab and ipilimumab. In the NSCLC subgroup, pembrolizumab was associated with a higher risk of high-grade AEs than nivolumab. In addition, ICIs (nivolumab, atezolizumab, and avelumab) led to a lower risk of any/high-grade treatment-related AEs than traditional chemotherapy and ICI combination chemotherapy. However, ICIs did not present preferable safety and tolerability compared to chemotherapy in treating melanoma. Compared with chemotherapy, nivolumab, durvalumab, two ICIs, and ICI combined chemotherapy led to more pneumonitis/pneumonia. However, when treating NSCLC, different types of ICIs did not differ significantly regarding the incidence of pneumonitis/pneumonia. A combination of nivolumab and ipilimumab had the highest risk for colitis, while pembrolizumab and atezolizumab had a lower possibility than the other ICIs.

**Conclusion:** Atezolizumab 1,200 mg and pembrolizumab 2 mg/kg every 3 weeks were ordinarily safer than other ICIs. When treating NSCLC, nivolumab had the lowest risk; when treating melanoma, pembrolizumab had the lowest toxicity.

## Introduction

Since ipilimumab, an anti–cytotoxic T lymphocyte-associated antigen 4 (CTLA-4) therapy, was approved by the Food and Drug Administration (FDA) in 2011, remarkable progress has been made in immunotherapy. As the first approved checkpoint inhibitor, ipilimumab is indicated only for melanoma. Another checkpoint inhibitor against the programmed death 1(PD-1)/programmed death ligand 1 (PD-L1) has also shown prominent success for patients with advanced solid tumors. In the National Comprehensive Cancer Network guidelines for non–small cell lung cancer (NSCLC) (Ettinger et al., 2017), an update focusing on targeted therapies and immunotherapies has been added to change the recommended therapy. The FDA suggested pembrolizumab as a first-line treatment for patients with PD-L1 expression levels ≥50% based on Keynote-024 (Brahmer et al., 2017). The indications of PD-1/PD-L1 were amplified after numerous clinical trials were completed and reported. Among these inhibitors, nivolumab and pembrolizumab alone and in combination with other agents have obtained approval by the FDA for melanoma and NSCLC monotherapy. Currently, ongoing clinical trials are focused on both PD-1 (nivolumab and pembrolizumab) and PD-L1 (atezolizumab, durvalumab, and avelumab) for different indications.

Compared with standard chemotherapy, immune checkpoint inhibitors (ICIs) showed great clinical benefits in prolonging the overall survival and progression-free survival for patients with solid tumors (Borghaei et al., 2015; Herbst et al., 2016; Sharma et al., 2016; Rittmeyer et al., 2017). This result was also indicated by evidence-based medical research (Zoratti et al., 2019; Frederickson et al., 2019). Along with the prominent efficacy of ICIs, adverse events (AEs) are gradually becoming concerns. In comprehensive real-world clinical use, chemotherapy has been clearly established as a general treatment with unequivocal benefits and survival advantages. Compared with traditional chemotherapy, ICIs can be taken as new administrations for advanced cancers with less toxicity and AEs. When the efficacy data on survival outcomes are reported in clinical trials and real-world practices, the understanding of the toxicities of immunotherapy needs to be expanded to establish better treatment options for advanced cancers. As inhibitors of immune checkpoints, CTLA-4 and PD-1/PD-L1 normally prevent the overactivation of the immune system and maintain the immune balance inside the body (Pardoll, 2012). This immune mechanism results in the toxicity reaction known as immune-related AEs, and classical chemotherapy toxicities also happen during treatment. Most AEs occur acutely and can be treated with steroids in 1 to 7 days (Johnson et al., 2018).

Acknowledging the AEs caused by ICIs is necessary for better clinical management. In a study by Wang W. et al. (2017), the risk of hepatotoxicity related to ICIs was demonstrated. Wang W. et al. (2017) reported that CTLA-4 inhibitors may lead to a high risk of hepatotoxicity, while PD-1 inhibitors had a low risk. The study by Nishijima et al. (2017) systematically reviewed the safety and tolerability of PD-1/PD-L1 inhibitors in advanced cancer and concluded that PD-1/PD-L1 inhibitors were overall better tolerated than chemotherapy. However, these studies did not compare the total immune-related or any treatment-related AEs. Direct meta-analyses were limited to the control group, which might overlook safety comparisons among different control arms in different clinical trials. Therefore, in this research, we conducted a systematic review and a network meta-analysis of randomized controlled trials (RCTs) to evaluate the AEs and toxicity among various ICIs and standard chemotherapy. As a previous trial conducted by Hellmann et al. (2018) showed, a combination of ipilimumab and nivolumab had a high response rate for NSCLC. However, it is difficult to acquire an integrated picture of AEs from RCTs when ICIs are indicated in two different cancers.

The purpose of this study was to systematically review and conduct a network meta-analysis on the safety and toxicity of different ICIs in treating NSCLC and melanoma. The risks for select specific treatment-related AEs (colitis and pneumonitis/pneumonia) were also compared among these different treatment patterns.

## Methods

### Systematic Review

The present report was performed according to the Preferred Reporting Items for Systematic Reviews and Meta-Analyses (PRISMA) guidelines and the PRISMA extension statement for network meta-analysis (Hutton et al., 2015). Two authors searched PubMed, EMBASE, Cochrane Library, and ClinicalTrials.gov independently for articles published between January 2000 and January 2019 with the following MeSH terms: “CTLA-4,” “PD-1,” “PD-L1,” “ipilimumab,” “atezolizumab,” “nivolumab,” “durvalumab,” “pembrolizumab,” and “avelumab” (**Supplement Figure 1**). Only RCTs were included. We also searched abstracts from the American Society of Clinical Oncology, and abstracts without full text were eliminated. The two reviewers assessed the screening results and made the final inclusion decisions. The references of relevant studies were also reviewed to include additional studies.

### Study Selection

Only randomized controlled clinical trials were included. Articles that met the following criteria were included: (a) phase II or phase III clinical trials on patients with NSCLC or melanoma; (b) studies with outcomes reporting of the rates of any all-grade and high-grade (3–4) AEs or treatment-related AEs that led to discontinuation or treatment-related death; and (c) at least one ICI as the intervention. It has been proven that autoimmune AEs occur, such as colitis, pneumonitis, skin AEs, endocrine dysfunction, and hepatitis (Johnson et al., 2018). It was also observed that, when treated with ipilimumab, patients had a higher risk for colitis than when treated with PD-1/PD-L1 inhibitors (Wang D. et al., 2017). The incidence of ICI-related pneumonia was also higher in the treatment of NSCLC than in the treatment of melanoma (Nishino et al., 2016). To explore the differences between the incidences of colitis and pneumonitis/pneumonia when patients were treated with ICIs, subgroup analyses of these two select AEs were conducted.

### Data Extraction

Two researchers (Q-QC and J-YD) independently conducted the data extraction. The following data were summarized: first author, title, year of publication, study ID, tumor site, trial phase, treatments, median follow-up time, version of the Common Terminology Criteria for Adverse Events, any AEs, treatment-related AEs, specific AEs, specific treatment-related AEs, treatment-related AEs leading to discontinuation, and treatment-related deaths.

### Quality Assessment

The qualities of the trials were ranked by the Jadad scale based on the original article, updated references and **Supplementary Materials** (**Figure 2**), the presence of sequence generation, allocation concealment, blinding, and incomplete and selective reporting (Jadad et al., 1996). When assessing the quality, a score of 2 was assigned for appropriate random sequence generation, accurate allocation concealment, and an appropriate description of blinding, and a score of 1 was assigned when there was incomplete and selective reporting. All disagreements in the study selection, data extraction, and quality assessment were discussed for consistency.

### Statistical Analysis

The primary objective of this article was to compare the toxicity and AEs among all ICIs and standard chemotherapy. Additionally, the differences in AEs between patients with NSCLC and melanoma were studied. Pairwise meta-analysis (PWMA) was applied for direct evidence that was pooled in random-effects models if heterogeneity existed (*P* < 0.05).

A total network was built containing all the included trials, and both direct and indirect comparisons were conducted. The consistency between the direct and indirect evidence was statistically confirmed by node-splitting analyses. The incidence of specific treatment-related toxicity, relative risk (RR) for any AEs, and odds ratio for high-grade AEs were calculated with 95% confidence intervals. When treatment-related AEs were not observed in the original studies, a relative index of any AEs that occurred during treatment was taken as a replacement. Heterogeneity among the trials was verified by the Cochrane Q statistic and quantified with the *I* ^2^ index (Higgins et al., 2003). When eminent heterogeneity was not shown (*P* > 0.05), pooled odds ratios/RRs and their 95% confidence intervals were reported in a fixed-effects model; otherwise, a random-effects model was applied.

Subgroups were created based on the cancer site, specific treatment-related AEs, and different ICIs. All analyses involved the use of the packages “gemtc” and “pcnetmeta” in R v3.5.1, and PWMA was conducted in Review Manager v5.3.

## Results

### Search Results and Eligible Trials

**Figure 1:**
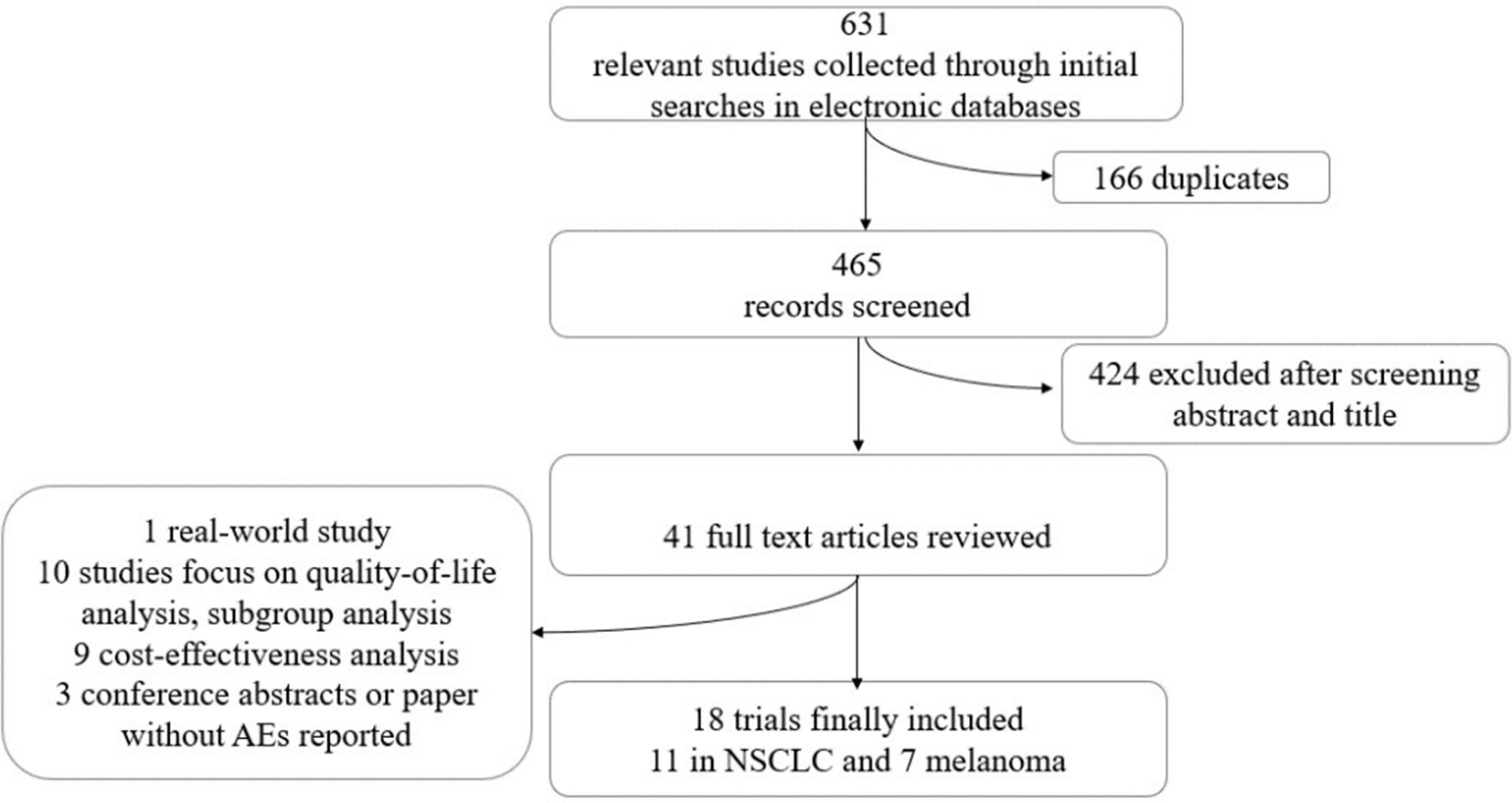
Flowchart of select of included trials in network meta-analysis.

The selection and exclusion criteria of the study are presented in **Figure 1**. A total of 631 studies were identified, of which 41 potential articles were reviewed intensively as full text. Finally, 18 randomized clinical trials, with a total of 11,223 patients, were incorporated in this network meta-analysis. In total, 11,018 patients had reported AE analyses in these original studies. The characteristics of these 18 trials are demonstrated in **Table 1**, among which 11 RCTs (Borghaei et al., 2015; Brahmer et al., 2015; Fehrenbacher et al., 2016; Herbst et al., 2016; Rittmeyer et al., 2017; Barlesi et al., 2018; Gandhi et al., 2018; Paz-Ares et al., 2018; Socinski et al., 2018; Antonia, 2019) compared ICIs to treat NSCLC, and seven trials (Larkin et al., 2015; Postow et al., 2015; Ribas et al., 2015; Robert et al., 2015; Schachter et al., 2017; Weber et al., 2017; Larkin et al., 2018) focused on melanoma. Nivolumab was used in eight trials, and the most common dosage was 3 mg/kg every 2 weeks intravenously. Another strategy was combining nivolumab 1 mg/kg with ipilimumab 3 mg/kg. Five RCTs containing pembrolizumab compared 2 or 10 mg/kg every 2 or 3 weeks with standard chemotherapies. Ribas et al. (2015), Herbst et al. (2016), and Schachter et al. (2017) also explored the outcomes when the dosage changed. Ipilimumab was indicated only for melanoma, and Larkin et al. (2015) and Postow et al. (2015) compared ipilimumab in different dosages with ipilimumab combined with nivolumab. Atezolizumab 1,200 mg was compared with docetaxel or used in combination therapy to treat NSCLC (Fehrenbacher et al., 2016; Rittmeyer et al., 2017; Socinski et al., 2018).

**Table 1:** Characteristics of 18 studies.

	Author, year	ID	Trial phase	Masking	Total N	Follow-up time (mo)	Inventions	Analyzed patients	CTCAE version	Discontinuation^*^
NSCLC										
1	(Brahmer et al., 2015)	Checkmate017	III	Open-label	272	UK	Nivolumab 3 mg/kg q 2 weeks	135	4.0	4
							docetaxel 75 mg/m^2^ q 3 weeks	137		13
2	(Govindan et al., 2017)	Checkmate026	III	Open-label	541		Nivolumab 3 mg/kg q 2 weeks	267	4.0	26
						UK	Platinum-based chemotherapy q3 weeks	263		35
3	(Borghaei et al., 2015)	Checkmate057	III	Open-label	582	14.5	Nivolumab 3 mg/kg q 2 weeks	287	4.0	14
							Docetaxel 75 mg/m^2^ q 3 weeks	268		40
4	(Rittmeyer et al., 2017)	OAK	III	Open-label	850	21	atezolizumab 1,200 mg	425	4.0	46
							docetaxel 75 mg/m^2^ q 3 weeks	425		108
5	(Fehrenbacher et al., 2016)	POPLAR	II	Open-label	287	14.8	Atezolizumab 1,200 mg	144		2
							Docetaxel 75 mg/m^2^ q 3 weeks	143		24
6	(Socinski et al., 2018)	IMPOWER150	III	Open-label	787	15.4	Atezolizumab + bevacizumab + carboplatin plus paclitaxel (ABCP)	393	4.0	128
							Bevacizumab + carboplatin + paclitaxel (BCP group)	394		98
7	(Herbst et al., 2016)	Keynote010	II/III	Open-label	1,034	10.4	Pembrolizumab 2 mg/kg q 3 weeks	339	4	15
							Pembrolizumab 10 mg/kg q 3 weeks	343		17
							Docetaxel 75 mg/m^2^ q 3 weeks	309		31
8	(Gandhi et al., 2018)	Keynote189	III	Double-blind	616	10.5	Pembrolizumab + pemetrexed + platinum-based drug	405	4	112
							Placebo + pemetrexed + Platinum-based drug	202		30
9	(Paz-Ares et al., 2018)	Keynote407	III	Double-blind	559	7.8	Pembrolizumab 200 mg + chemotherapy	278	4.03	37
							Placebo + chemotherapy	280		34
10	(Antonia et al., 2017)	PACIFIC	III	Double-blind	713	14.5	Durvalumab 10 mg/kg q 2 weeks	475	4.03	73
							Placebo	234		23
11	(Barlesi et al., 2018)	JAVELIN Lung 200	III	Open-label	792	18.3	Avelumab 10 mg/kg q 2 weeks	393	4.03	28
							Docetaxel 75 mg/m² q 3 weeks	365		51
Melanoma										
12	(Larkin et al., 2018)	Checkmate037	III	Open-label	405	24	Nivolumab 3 mg/kg q 2 weeks	268	4.0	13
							Chemotherapy	102		11
13	(Robert et al., 2015)	Checkmate066	III	Double-blind	418	16.7	Nivolumab 3 mg/kg q 2 weeks	206	4.0	14
							Dacarbazine 1,000 mg/m^2^ q 3 weeks	205		24
14	(Larkin et al., 2015)	Checkmate067	III	Double-blind	945	9	Nivolumab 1 mg/kg + ipilimumab 3 mg/kg	313	4.0	24
							Nivolumab 3 mg/kg q 2 weeks	313		114
							Ipilimumab 3 mg/kg q 3 weeks	311		46
15	(Postow et al., 2015)	Checkmate069	II	Double-blind	142	24.6	Nivolumab 1 mg/kg + Ipilimumab 3 mg/kg	94	4.0	44
							Ipilimumab 3 mg/kg q 3 weeks	46		8
16	(Weber et al., 2017)	Checkmate238	III	Double-blind	906	19.5	Nivolumab 3 mg/kg q 2 weeks	452	4.0	35
							Ipilimumab 10 mg/kg q 3 weeks	453		189
17	(Ribas et al., 2015)	Keynote 002	II	Double-blind	540	10	Pembrolizumab 2 mg/kg q 3 weeks	180	4.0	4
							Pembrolizumab 10 mg/kg q 3 weeks	181		13
							Chemotherapy	179		10
18	(Schachter et al., 2017)	Keynote006	III	Open-label	834	22.9	Pembrolizumab 10 mg/kg, q 2 weeks	278	4.0	19
							Pembrolizumab 10 mg/kg, q 3 weeks	277		30
							Ipilimumab q 3 weeks	256		23

*Discontinuation for treatment-related Aes CTCAE, Common Terminology Criteria for Adverse Events; UK, unknown; q 2 weeks, every 2 weeks; q 3 weeks, every 3 weeks.

Detailed characteristics of the included trials are shown in **Table 1**. The modified Jadad scores indicated that almost all data included in this network meta-analysis (NMA) were from high-quality studies, with only one study that had the lowest score of 3. All trials were randomly designed, but only eight (44.44%) demonstrated the generation of random sequences, and there was no selective or incomplete outcomes reporting.

### Network Geometry

**Figure 2:**
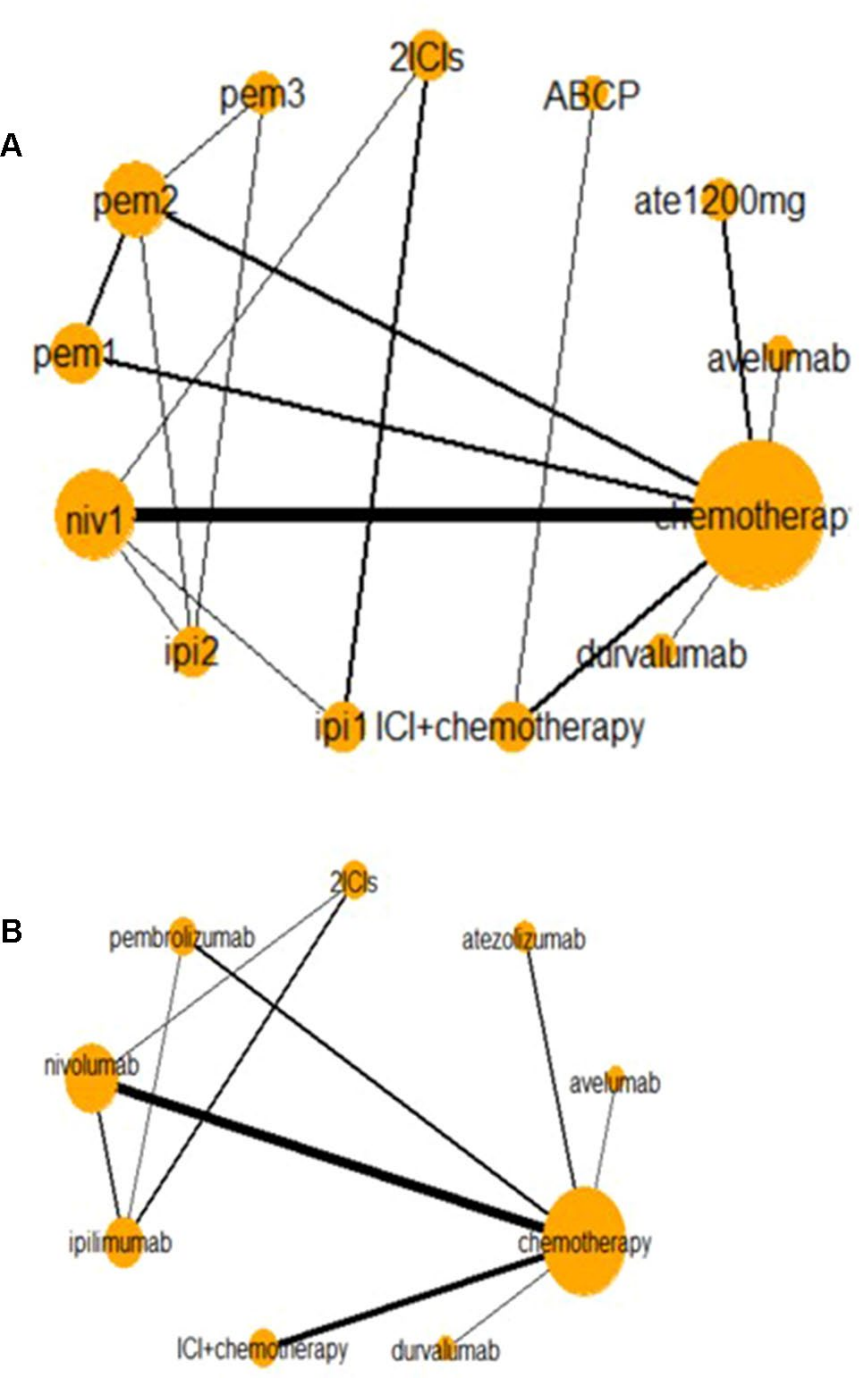
Network of all trials **(A)** and ICIs combined **(B)** for the Bayesian network meta-analysis. Each node presents an invention in the trial. Size of node is proportional to the number of patients. niv1, nivolumab 3 mg/kg, q 2 weeks; pem1, pembrolizumab 2 mg/kg, q 3 weeks; pem2, pembrolizumab 10 mg/kg, q 3 weeks; pem3, pembrolizumab 10 mg/kg, q 2 weeks; ipi1, ipilimumab 3 mg/kg, q 3 weeks; ipi2, ipilimumab 10 mg/kg, q 3 weeks; ABCP, atezolizumab + bevacizumab + carboplatin + paclitaxel. Relative risk (RR) and odds ratio (OR) with 95% confidence interval (CI) in bold means it is statistically significant when comparing these two groups. And values <1 favor the intervention group instead of the control group. For instance, when comparing nivolumab and chemotherapy in high-grades AEs, OR with 95% CI [0.42 (0.20–0.86)] means that fewer AEs happen in intervention group (nivolumab), and it is statistically significant. And when comparing any-grade treatment-related AEs in nivolumab and chemotherapy, RR with 95% CI [1.09 (0.98–1.31)] suggests that fewer AEs happen in the control group (nivolumab), but it is not statistically significant.

**Figure 2** presents two network diagrams illustrating the whole network: a total network meta-analysis and a comparison among different ICIs. The cancer-based analysis is presented in **Supplement Figure 3**. Chemotherapy was the most common control group, and this group had the largest proportion of patients.

### Network Meta-Analysis for Treatment-Related AEs

All relative outcomes of any-grade or high-grade treatment-related AEs in the NMA are presented in **Supplement Figure 4**. Compared with chemotherapy, nivolumab 3 mg/kg, atezolizumab 1,200 mg, and pembrolizumab 2 or 10 mg/kg every 3 weeks had a lower risk of high-grade AEs. When ICI was combined with chemotherapy, the risk of suffering from high-grade treatment-related AEs was higher than that with nivolumab 3 mg/kg, atezolizumab 1,200 mg, pembrolizumab 2 or 10 mg/kg, ipilimumab 3 mg/kg every 3 weeks, or avelumab 10 mg/kg every 2 weeks. This finding might imply that monotherapy with some ICIs was more tolerable than ICI combination chemotherapy, but there was no evidence of superiority between chemotherapy and combination therapy. In the comparison of ICI combination chemotherapy with nivolumab 1 mg/kg plus ipilimumab 3 mg/kg, no significant differences were observed.

**Figure 3:**
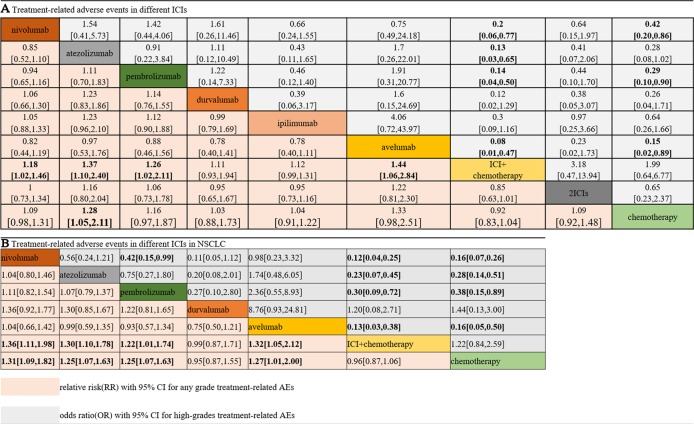
Safety and tolerance of different ICIs in network meta-analysis in consistency model. **A**: treatment-related adverse events in different ICIs; **B**: treatment-related adverse events in different ICIs for NSCLC subgroup.

**Figure 3A** shows the results of the network meta-analysis based on different ICIs. The network meta-analysis demonstrated a significantly higher risk of all AEs with ICI plus chemotherapy than with nivolumab, atezolizumab, pembrolizumab, and avelumab. In other words, monotherapy with ICIs led to a lower risk of suffering from AEs than combination therapy with any ICIs, except durvalumab or ipilimumab, and this finding was consistent with the outcomes regarding high-grade treatment-related AEs. In the analysis of high-grade AEs, nivolumab and pembrolizumab were more tolerable than chemotherapy, regardless of dosage. The safety ranking for any-grade AEs is as follows: avelumab (40%), atezolizumab (32%), pembrolizumab (22%), nivolumab (23%), ipilimumab (21%), nivolumab plus ipilimumab (11%), chemotherapy (46%), durvalumab (20%), and ICI plus chemotherapy (71%); this ranking was mainly the same as the ranking for high-grade AEs. The possibility of avelumab becoming the safest ICI was 40%, and ICI plus chemotherapy had a 71% probability of being the least tolerant.

### Subgroup Analysis Between NSCLC and Melanoma

The patients were divided into NSCLC and melanoma subgroups. Group NSCLC involved 11 original studies with 7,033 patients, while the melanoma group involved 4,190 patients from seven articles. **Figure 3B** shows that the risk of both any-grade and high-grade treatment-related AEs was lower with nivolumab, atezolizumab, and avelumab than with ICI combination chemotherapy and traditional chemotherapy. Pembrolizumab was superior to ICI combination chemotherapy but not to traditional chemotherapy. The results for high-grade AEs remained roughly identical with those for any-grade AEs, with the exception of pembrolizumab. Pembrolizumab also showed a lower risk than traditional chemotherapy, but pembrolizumab was related to a higher risk of high-grade AEs than nivolumab. Unexpectedly, durvalumab showed intolerability in terms of high-grade AEs, even more so than ICI combination chemotherapy. In the melanoma subgroup, ICIs did not show better safety or more tolerability than chemotherapy, which is different from the outcomes of the NSCLC subgroup.

### Pneumonitis/Pneumonia and Colitis as Treatment-Related AEs

In the selected AE analyses, indirect comparisons were conducted on pneumonitis/pneumonia and colitis. The results suggested that nivolumab, durvalumab, two ICIs, and ICI combination chemotherapy would remarkably increase the risk of any-grade pneumonitis/pneumonia compared with chemotherapy. Avelumab was the only ICI that might be ranked higher (lower risk) than chemotherapy. However, the risks did not vary in the NSCLC subgroup among different ICIs.

**Figure 4:**
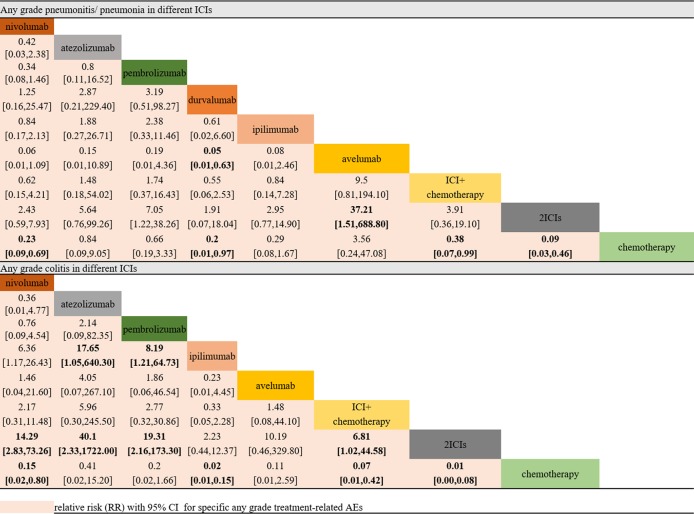
Selected immune-related any-grade AEs in different ICIs.

In the colitis analysis, ipilimumab and two ICIs (nivolumab + ipilimumab) had the highest risk of occurrence. In a sensitive analysis that ignored durvalumab and did not report the risk of colitis, we found that nivolumab combined with ipilimumab could cause more colitis than other ICIs. In general, pembrolizumab and atezolizumab had a lower possibility of leading colitis than other ICIs. All the outcomes are shown in **Figure 4**.

### Inconsistency Assessment and Sensitivity Analysis

The node-splitting analysis indicated no significant inconsistencies except for the comparison between nivolumab and two ICIs (**Supplement Figure 5**). Two groups of PWMAs were included, taking chemotherapy and ipilimumab as the control groups (**Table 2**). The direct evidence indicated that atezolizumab, pembrolizumab, and avelumab showed a lower risk of any- or high-grade AEs than other ICIs. Nivolumab was only superior to other ICIs for high-grade AEs. Heterogeneity between groups was found for the comparisons of nivolumab versus chemotherapy and nivolumab versus ipilimumab (*I* ^2^ > 50%, *P* < 0.05). For the one direct comparison, obvious inconsistency existed between the network meta-analysis and direct comparison for durvalumab, which presented a drastically higher risk than chemotherapy for any- and high-grade treatment-related AEs in the PWMA.

**Table 2:** Forest plot of direct and indirect results of head-to-head trials.

					Heterogeneity	
Inventions	Study/patients		RR/OR (95% CI)	P	*I* ^2^ (%)	P
Control: chemotherapy						
Nivolumab	5/2,138	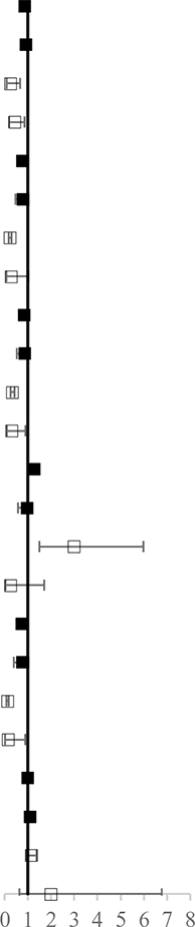	0.85 (0.69–1.04)	0.11	97	<0.00001
			0.92 (0.76–1.02)			
			0.25 (0.09–0.67)	0.006	95	<0.00001
			0.42 (0.20–0.86)			
Atezolizumab	2/1,474		0.76 (0.71–0.80)	<0.00001	0	0.46
			0.78 (0.47–0.96)			
			0.23 (0.18–0.30)	<0.00001	0	0.86
			0.28 (0.08–1.02)			
Pembrolizumab	2/1,531		0.84 (0.74–0.96)	0.009	73	0.05
			0.86 (0.54–1.03)			
			0.33 (0.26–0.43)	<0.00001	0	0.41
			0.29 (0.10–0.90)			
Durvalumab	1/447		1.27 (1.11–1.45)	0.0005	NA	NA
			0.97 (0.58–1.13)			
			2.99 (1.50–5.98)	0.002	NA	NA
			0.26 (0.04–1.71)			
Avelumab	1/564		0.74 (0.68–0.81)	<0.00001	NA	NA
			0.75 (0.40–1.03)			
			0.12 (0.08–0.18)	<0.00001	NA	NA
			0.15 (0.02–0.89)			
ICI + chemotherapy	3/1,952		1.00 (0.98–1.01)	0.67	35	0.21
			1.09 (0.96–1.20)			
			1.14 (0.94–1.38)	0.17	0	0.76
			1.99 (0.64–6.77)			
Control: ipilimumab						
2 ICIs	2/276	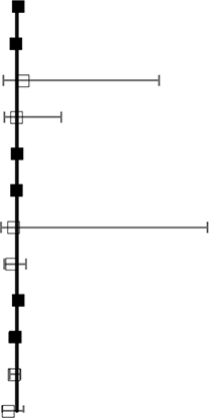	1.05 (0.93–1.19)	0.44	80	0.03
			0.95 (0.73–1.16)			
			1.36 (0.19–9.52)	0.76	95	<0.00001
			0.97 (0.25–3.66)			
Nivolumab	2/1,529		0.99 (0.80–1.23)	0.94	98	<0.00001
			0.95 (0.75–1.13)			
			0.80 (0.05–12.42)	0.87	99	<0.00001
			0.66 (0.24–1.55)			
Pembrolizumab	1/811		1.08 (0.99–1.17)	0.09	NA	NA
			0.89 (0.53–1.11)			
			0.83 (0.57–1.21)	0.34	NA	NA
			0.46 (0.12–1.40)			

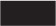
 RR in any-grade treatment-related AEs. 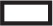 OR in high-grade treatment-related AEs.

Upper is network analysis; below is PWMA. CI, confidence interval; OR, odds ratio; RR, relative risk.

## Discussion

As the number of FDA approvals for ICIs increases, the indications for different ICIs have also expanded. However, different ICIs have distinct immunologic mechanisms and should not be taken as a whole category; even ICIs that belong to the same mechanism might lead to unlikely treatment effects and tolerability in different diseases (Sukari et al., 2019). This review included 18 phase II/III clinical trials, which involved 11,223 patients suffering from NSCLC and melanoma. In the analysis of all included trials, 10 mg/kg avelumab every 2 weeks was considered the most tolerable, and 1,200 mg atezolizumab was ranked second. When treating NSCLC, nivolumab was ranked as having the lowest risk for both any- and high-grade AEs, followed by avelumab. In the subgroup for melanoma, pembrolizumab was superior to nivolumab, ipilimumab, two combined ICIs, and chemotherapy. Chemotherapy and ICI combined with chemotherapy were ranked low in safety regardless of the dosage or cancer type. It was suggested that nivolumab and avelumab were safe options for NSCLC and pembrolizumab for melanoma regarding any-grade or high-grade AEs. However, due to the failure of avelumab in treating NSCLC (Barlesi et al., 2018), atezolizumab 1,200 mg and nivolumab were favorable choices.

Several meta-analyses and network meta-analyses concerning the safety and tolerability of ICIs have been reported (Nishijima et al., 2017; Baxi et al., 2018; Komaki et al., 2018; Xu et al., 2018; Su et al., 2019; Zoratti et al., 2019). These prior studies focused on simple solid tumors, and select immune-related AEs were also reported. Few of these studies made a comparison among all the inhibitors approved by FDA. In contrast, we comprehensively included all possible ICI regimens for treating NSCLC and melanoma. These two solid tumors were largely potential indications for immunotherapy, so such inhibitors would already be used.

In our analysis, most clinical trials used chemotherapy as a controlled arm, and we performed direct and indirect analyses to compare all types of ICIs, not only head-to-head trials. This process was different from that of a previous meta-analysis, which only contained direct comparisons. More importantly, pneumonia and colitis (two specific AEs related to ICI treatment) were analyzed among different ICIs. This study indicated that ICI leads to more pneumonitis/pneumonia and colitis than chemotherapy. Avelumab has the lowest risk for pneumonitis/pneumonia among all comparators, including chemotherapy. Compared with pembrolizumab and avelumab, the combination of two ICIs (nivolumab + ipilimumab) might lead to a higher risk of any-grade pneumonitis/pneumonia (**Figure 3**, RR > 1). However, no significant differences were observed among the monotherapy ICI regimens. Our findings suggested that there were no notable differences among different ICIs regarding the risk for pneumonitis/pneumonia, which was consistent with the study reported by Nishino et al. (2016). In summary, when treated with ICIs, patients with NSCLC would have a higher risk of pneumonitis/pneumonia than those with melanoma, but this difference was not related to the kind of ICI. In addition, a high correlation was observed between ipilimumab and colitis. Ipilimumab led to a higher risk for colitis than nivolumab, atezolizumab, or pembrolizumab. We also noted that nivolumab, ipilimumab, and the combination of these two ICIs would lead to a higher risk of any-grade colitis than chemotherapy. The combination of nivolumab and ipilimumab led to a higher risk for colitis than even one ICI combined with chemotherapy. In addition, colitis should be given more attention when nivolumab is administered, and pembrolizumab is the much safer option of the two in that aspect. Based on these comprehensive results, this evidence-based analysis might suggest that when nivolumab and ipilimumab are combined, there is concern of colitis. The differences between these two solid tumors might suggest that the specificity of immune-related AEs was closely associated with the mechanism of the ICIs.

The current analysis has several strengths. By comprehensively including the latest data up to January 2019, we considered all the available evidence on any treatment containing ICIs for NSCLC and melanoma. A detailed assessment of the credibility of the evidence was performed to appraise the results critically. Then, this network meta-analysis was conducted. First, we made a general comparison among all the direct and indirect evidence with different clinical dosages. Thus, a conclusion about the influence of dosage was drawn. Second, we considered any-grade and high-grade AEs from different ICIs to explore the discrepancy among those drugs. A PWMA was also conducted for a head-to-head comparison of the clinical trials of different ICIs. Third, subgroup analyses for NSCLC and melanoma showed different safety and tolerability. Finally, select specific AEs (pneumonitis/pneumonia and colitis) were reported in this review to identify the different immune-related effects.

Limitations also exist in this analysis. Due to the nature of network meta-analyses, missing values always exist in published articles. In the current analysis, we conducted a comprehensive assessment of the evidence we collected and excluded low-quality evidence to improve the quality of this review. Second, some treatments (durvalumab and avelumab) were adopted in only one clinical trial, which might lead to a biased evaluation without enough head-to-head evidence. Third, as the reported AE types were different among the original trials, the specific treatment-related AEs could not be completely evaluated. Thus, we focused on any-grade and high-grade treatment-related AEs as the primary outcome, which could suggest the overall safety and tolerability. Additionally, specific AEs related to ICIs for NSCLC and melanoma were selected to distinguish the differences between tumor types. Third, the incidences of immune-related AEs (including pneumonitis/pneumonia and colitis) were not high, especially those of serious lung toxicities and colitis (Johnson et al., 2018). The low incidence may substantially influence the final results of the indirect comparisons. The influence would be particularly obvious if the specific AE was not reported in the original study. Fourth, this research did not consider the impact of the different systemic therapies before ICI treatment and the expression level of PD-L1, which might imply inevitable heterogeneity among the included trials.

## Conclusion

In summary, atezolizumab 1,200 mg and pembrolizumab 2 mg/kg every 3 weeks were generally safer than other ICIs. Nivolumab and pembrolizumab were safer for NSCLC and melanoma than other ICIs, respectively.

## Author Contributions

Q-QC is the first author of this NMA, and she is responsible for the modification of this paper. J-YD is the second author. JZ is the third author. BW is the corresponding author, and he takes responsibility for the authenticity of the paper and also the modification.

## Funding

This work was sponsored by unrestricted grants from the National Natural Science Foundation of China (NO. 7172810).

## Conflict of Interest

The authors declare that the research was conducted in the absence of any commercial or financial relationships that could be construed as a potential conflict of interest.
