# Supplementary material for: The Differences in the Safety and Tolerability of Immune Checkpoint Inhibitors as Treatment for Non–Small Cell Lung Cancer and Melanoma: Network Meta-Analysis and Systematic Review
Source: Front Pharmacol. 2019 Oct 24;10:1260. doi: 10.3389/fphar.2019.01260 (PMC6821878; doi:10.3389/fphar.2019.01260)
Supplement: Supplementary file 1 [file Table_1.docx]

**Supplement materials**

S1 Details of Research Strategy in Online Database

**PubMed**

**#NSCLCL** Search **(((NSCLC[MeSH Terms]) OR non-small cell lung cancer)) AND (((((((((((((((nivolumab[MeSH Terms]) OR nivolumab) OR pembrolizumab[MeSH Terms]) OR pembrolizumab) OR atezolizumab[MeSH Terms]) OR atezolizumab) OR durvalumab[MeSH Terms]) OR durvalumab) OR avelumab[MeSH Terms]) OR avelumab) OR ipilimumab[MeSH Terms]) OR ipilimumab) OR CTLA-4[MeSH Terms]) OR PD-1) OR PD-L1)** Sort by: **Best Match** Filters: **Clinical Trial; Publication date from 2000/01/01 to 2019/01/01**

**#Melanoma** Search **(((melanoma[MeSH Terms]) OR melanoma)) AND (((((((((((((((nivolumab[MeSH Terms]) OR nivolumab) OR pembrolizumab[MeSH Terms]) OR pembrolizumab) OR atezolizumab[MeSH Terms]) OR atezolizumab) OR durvalumab[MeSH Terms]) OR durvalumab) OR avelumab[MeSH Terms]) OR avelumab) OR ipilimumab[MeSH Terms]) OR ipilimumab) OR CTLA-4[MeSH Terms]) OR PD-1) OR PD-L1)** Sort by: **Best Match** Filters: **Clinical Trial; Publication date from 2000/01/01 to 2019/01/01**

**Embase**

**#NSCLC** 'lung non-small cell carcinoma cell line'/exp AND ('pembrolizumab'/exp OR 'nivolumab'/exp OR 'atezolizumab'/exp OR 'durvalumab'/exp OR 'avelumab'/exp OR 'ipilimumab'/exp OR 'cytotoxic t lymphocyte antigen 4'/exp OR 'programmed death 1 receptor'/exp OR 'pd l1 antibody'/exp) AND [1-1-2000]/sd NOT [2-1-2019]/sd

**#Melanoma** 'melanoma'/exp AND ('pembrolizumab'/exp OR 'nivolumab'/exp OR 'atezolizumab'/exp OR 'durvalumab'/exp OR 'avelumab'/exp OR 'ipilimumab'/exp OR 'cytotoxic t lymphocyte antigen 4'/exp OR 'programmed death 1 receptor'/exp OR 'pd l1 antibody'/exp) AND 'randomized controlled trial'/exp AND [1-1-2000]/sd NOT [2-1-2019]/sd

Figure2 Quality Assessment of Included Trials Using Modified Jadad Score

| study |  | | quality assessment | | | | | |
| --- | --- | --- | --- | --- | --- | --- | --- | --- |
|  | trial name | random sequence generation | | allocation concealment | blinding | complete outcome data | no selective reporting | modified Jadad score |
| NSCLS |  |  | |  |  |  |  |  |
| Julie Brahmer,2015 | CheckMate017 | UN | | UN | N | Y | Y | 3 |
| D.P. Carbone2017 | CheckMate026 | UN | | N | Y* | Y | Y | 5 |
| H. Borghaei2015 | CheckMate057 | UN | | N | Y* | Y | Y | 5 |
| Achim Rittmeyer2016 | OAK | Y | | Y | N | Y | Y | 6 |
| Louis Fehrenbacher2016 | POPLAR | Y | | Y | N | Y | Y | 6 |
| M.A. Socinski2018 | IMPOWER150 | UN | | UN | N | Y | Y | 4 |
| Roy S Herbst2016 | Keynote010 | UN | | UN | N | Y | Y | 4 |
| L. Gandhi2018 | Keynote 189 | Y | | Y | Y | Y | Y | 7 |
| L. Paz‑Ares2018 | Keynote 407 | Y | | Y | Y | Y | Y | 7 |
| S.J. Antonia2017 | PACIFIC | UN | | UN | Y | Y | Y | 5 |
| Fabrice Barlesi2018 | JAVELIN Lung 200 | Y | | Y | N | Y | Y | 6 |
| Melanoma |  |  | |  |  |  |  |  |
| James Larkin | CheckMate037 | UN | | UN | N | Y | Y | 4 |
| Caroline Robert | CheckMate 066 | UN | | UN | Y | Y | Y | 5 |
| James Larkin | CheckMate 067 | UN | | UN | Y | Y | Y | 5 |
| Michael A2017 | CheckMate 069 | Y | | Y | Y | Y | Y | 7 |
| J. Weber.2017 | CheckMate 238 | UN | | Y | Y | Y | Y | 6 |
| Antoni Ribas2015 | Keynote 002 | Y | | Y | Y | Y | Y | 7 |
| Jacob Schachter2017 | Keynote 006 | Y | | Y | N | Y | Y | 6 |
| Y*: chemotherapies were chosen by investigator for the different in the trial design, and it was clearly stated in design so it is still marked as Y in bias assessment. | | | | | | | | |
| high quality: score ≥ 4 | | | |  |  |  |  |  |
| UN: illustration in the trial design was not clear | | | | | |  |  |  |

Figure3 Cancer-based Network Geometry Analysis

1. NSCLC


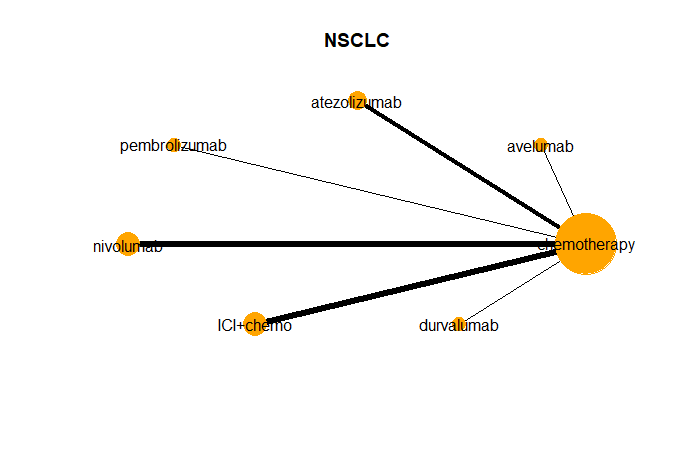


1. Melanoma


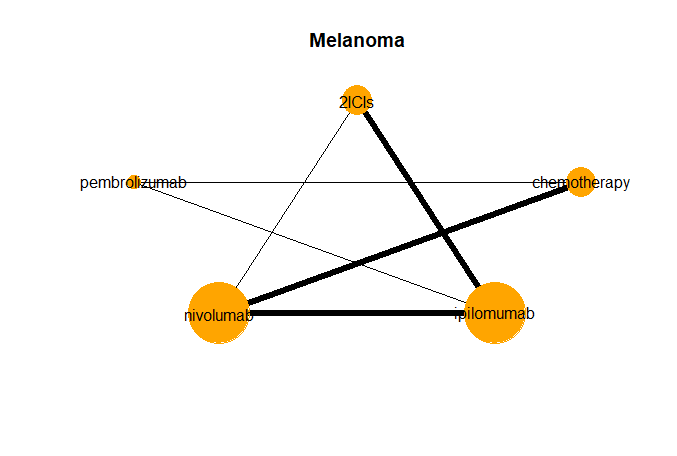


Figure4 High-grades Treatment-related AEs in All Inventions Included

Figure5 Node-splitting Analysis

| Node-splitting analysis of inconsistency in any grade and high-grades AEs in ICIs | | | | | | |
| --- | --- | --- | --- | --- | --- | --- |
| Comparison | | Any grade | | | High-grades | |
|  | | p value | 95%CI |  | p value | 95%CI |
| nivolumab vs durvalumab | | 0.8069 |  |  | 0.8587 |  |
| direct |  |  | 0.093 (-1.5, 1.7) | |  | 0.23 (-1.5, 2.0) |
| indirect |  |  | 0.28 (-3.2, 2.5) | |  | 0.52 (-2.7, 3.8) |
| network |  |  | -0.028 (-1.3, 1.2) | |  | 0.017 (-1.5, 1.6) |
| nivolumab vs 2ICIs | | 0.2085 |  |  | 0.0131 |  |
| direct |  |  | -1.6 (-3.4, 0.27) | |  | -1.8 (-3.8, 0.10) |
| indirect |  |  | 0.43 (-2.3, 3.0) | |  | 2.6 (0.033, 5.2) |
| network |  |  | -0.75 (-2.4, 0.87) | |  | -0.18 (-2.2, 1.8) |
| nivolumab vs chemotherapy | | 0.8331 |  |  | 0.7257 |  |
| direct |  |  | 0.68 (-0.35, 1.6) | |  | 1.4 (0.17, 2.6) |
| indirect |  |  | 0.98 (-2.0, 3.9) | |  | 0.76 (-3.0, 4.6) |
| network |  |  | 0.72 (-0.22, 1.6) | |  | 1.3 (0.23, 2.4) |
| pembrolizumab vs ipilimumab | | 0.8371 |  |  | 0.7260 |  |
| direct |  |  | -0.31 (-2.4, 1.8) | |  | 0.18 (-2.5, 2.9) |
| indirect |  |  | -0.014 (-2.3, 2.4) | |  | -0.45 (-3.4, 2.5) |
| network |  |  | -0.19 (-1.7, 1.3) | |  | -0.11 (-2.0, 1.8) |
| pembrolizumab vs chemotherapy | | 0.8298 |  |  | 0.7248 |  |
| direct |  |  | 0.63 (-0.89, 2.1) | |  | 1.1 (-0.84, 3.0) |
| indirect |  |  | 0.32 (-2.5, 3.0) | |  | 1.7 (-1.7, 5.2) |
| network |  |  | 0.56 (-0.69, 1.8) | |  | 1.2 (-0.37, 2.8) |
